# Unveiling the shadow economy in emerging markets

**DOI:** 10.1371/journal.pone.0347882

**Published:** 2026-05-13

**Authors:** Quynh Nga Duong, Nguyen Thuy Khue Tran

**Affiliations:** 1 Department of Air Transport Economics, Vietnam Aviation Academy, Hochiminh City, Vietnam; 2 Deparment of Business Administration, Vietnam Aviation Academy, Hochiminh City, Vietnam; Visva-Bharati University: Visva-Bharati, INDIA

## Abstract

This study examines the direct role of the shadow economy in economic growth and its indirect roles through interaction with tax burden, institutional environment, foreign direct investment, trade openness, government consumption, and population growth. Although shadow economy has been comprehensively studied in the literature, both joint and environment-specific effects for emerging economies are not well documented. This research fills this void by integrating several dimensions of structure into one Bayesian MCMC model. The Bayesian approach is used because it is powerful in capturing parameter uncertainty and interaction that is challenging to model under limited data. Empirical evidence from a balanced panel of developing countries indicates that the shadow economy reduces per capita GDP, but such adverse impacts are dampened under strong institutions quality, higher foreign direct investment, and higher trade openness. Conversely, higher population growth and over government consumption reinforce its adverse impacts. The findings present a new, interaction-oriented account of shadow economy and contend that institutional change and good fiscal policy are essential in diminishing informality and promoting inclusive and sustainable growth.

## 1. Introduction

The shadow economy, comprised of concealed, untaxed, or unregistered endeavors, is a widespread feature of most emerging economies wherein considerable portions of work and production take place beyond formal institutions [1]. While shadow economy can catalyze entrepreneurship and provide income prospects for marginalized groups, it also erodes fiscal capacity, governance, and exacerbates inequality—at the expense of jeopardizing long-run sustainable development [2].

Despite its importance, the manner in which the shadow economy interacts with structural and institutional determinants— such as institutional quality, foreign direct investment (FDI), and fiscal policy— remains unclear [3]. Weak institutions can allow shadow economy to persist, while FDI and trade openness can formalize or entrench it based on the governance quality [4]. This unresolved paradox calls for a more cautious examination of the joint impact of these factors on growth outcomes in the developing world [5,6].

In this study, the relationship that exists between the shadow economy and economic growth will be explored using the dual economy theory [7] and the endogenous growth theory [8,9], where the moderating factor of interest will be the quality of institutions [10], along with FDI, trade openness, the tax burden, government consumption, and population growth [11,12].

To this end, this study answers three guiding questions:

What is the effect of the shadow economy on economic growth in emerging economies?

How do institutional and structural determinants moderate this relationship?

What are the policy interventions that can mitigate the adverse effect of informality?

To answer these questions, the paper employs a Bayesian Markov Chain Monte Carlo (MCMC) framework for developing countries’ panel data [13]. The Bayesian approach is particularly well suited to deal with parameter uncertainty, model complexity, and potential nonlinearities under limited or heterogeneous data [13,14]. The empirical results indicate that while the shadow economy has an overall negative impact on GDP per capita, this is mostly undone by good institutions, higher FDI, and higher trade openness [15]. Fiscal pressure and high population growth, however, reinforce informality’s drag on growth [16].

The paper’s significance lies in its ability to combine institutional factors, fiscal variables, and structural insights in a single framework of analysis to better understand the role of the shadow economy in the process of economic growth. The paper makes use of Bayesian econometrics to identify conditional and interaction effects that can be masked in a straightforward estimation framework. The model also presents novel findings from a policy framework concerning the role of governance quality, foreign direct investment, and fiscal policy in the interaction approach that determines the dynamics of the shadow economy and the development of the formal economy. The paper extends the development of sustainable development theory in emerging markets through this interaction approach.

The rest of the paper is organized as follows: Section 2 integrates the theoretical and empirical literature relevant to the investigation around composite themes for conciseness. Section 3 provides the theoretical model and hypotheses formulation, connecting the conceptual foundations to empirical testable hypotheses. Section 4 outlines the methodology, data, variables, and Bayesian estimation approach. Section 5 presents the findings, and Section 6 provides a full discussion of the results in the light of past research and theory. Section 7 concludes with recommendations for policy and future research.

## 2. Literature review

The shadow economy—consisting of concealed, untaxed, or unregistered economic activity—is a characteristic of emerging economies in which elevated shares of employment and business activity occur outside institutions [1–3]. Shadow economy is a blessing and a curse: it offers prospects for entrepreneurship and work for out-of-pocket workers but also detracts from fiscal capacity, fosters corruption, and increases inequality [2].

Shadow economy thus threatens sustainable development [4,5,17]. Unraveling the manner in which the shadow economy interacts with such underlying structural and institutional determinants is key to developing effective formalization policies.

Following dual economy and endogenous growth theory [6,7], the present research investigates the intricate interdependence between economic growth and the shadow economy with emphasis on their moderation by institutional quality, foreign direct investment (FDI), trade openness, tax burden, government consumption, and population growth. Existing research has mainly focused on developed economies, while developing economies, where informality is most rooted, remain relatively under-explored country group. Furthermore, previous research used to establish these determinants independent of one another, without taking into account how the determinants might interact with informality and affect growth outcomes in significant manners.

Consistent with this, this paper answers three fundamental questions:

- How does the shadow economy affect economic growth in developing economies?
- How do structural and institutional contexts mediate the effect?
- What are the policy considerations for averting informality’s detrimental impacts?

The study contributes by:

(1) Combining institutional, cultural, and structural dimensions within shadow economy;
(2) Employing a Bayesian MCMC framework to account for parameter uncertainty and nonlinearities in the case of limited data availability; and
(3) Revealing empirical evidence on the joint contributions of informality, governance, and macro fundamentals to growth in emerging markets.

With the help of an interaction-based informality account, this research extracts new implications for upgrading formalization, fiscal sustainability, and sustainable and inclusive growth.

### 2.1. Economic growth and the shadow economy

The shadow economy has long been a disputed topic among economists, with its contribution to growth being complex and context-dependent. Generally, there are two major views in the debate: the “sand the wheels” hypothesis, which considers the shadow economy a brake on growth, and the “grease the wheels” hypothesis, which suggests that it can grease economic growth in certain conditions [1].

In a negative context, the shadow economy is traditionally considered to be a stranglehold on normal economic growth [3]. It erodes the tax base, reduces government expenditures on essential infrastructure and education, and skews official statistics, which makes policymaking complicated [5]. High informality levels have the potential to deteriorate institutional quality, reduce the efficiency of public administration, and also increase corruption, all of which constrain the formal sector’s growth potential [18]. For example, Schneider and Enste (2000) identify high taxation and excessive bureaucracy as the principal drivers of informality, which subsequently reduces GDP growth that is registered. In countries like Tunisia, whose economies are dominated by informal-hybrid economies, the shadow economy has been shown to impede the ability of the financial sector to stimulate economic growth, thereby aggravating long-term growth challenges [19,20]. Similarly, the Colombian record 1980–2012 demonstrates this dynamic to work, with shadow economy equaling between 27% and 56% of GDP for those thirty years, and contributing to an average real per capita GDP growth rate of only 1.86%—slightly less than 0.12 percentage points that would have existed in their absence [21]. A nonlinear effect of the shadow economy has been observed to positively contribute to the growth of Malaysia’s economy through expansions of the shadow economy especially in the short term, according to research carried out by Gamal et al. in 2025 [22,23].

With some caveats, there are, however, positive effects to be gained. The informal economy can be a safety net, providing employment and income for those not within the formal economy, particularly in institutionally weak economies [24]. This can also promote entrepreneurial activity through reduced costs of entry and the ability to conduct business without being bound by formal regulatory requirements [25]. For example, as the dual economy model explains, the majority of the financial gains from the shadow economy are used to fund the formal economy, which has spillover effects that aid consumption expenditure [26]. In this regard, the shadow economy has the ability to contribute to the economic resilience of an economy during times of recession when the formal economy remains less developed [25].

Nevertheless, the net impact of the shadow economy regarding growth has been largely driven by the circumstances [25]. It depends on numerous factors, ranging from governance quality to the exercise of institutional power, the efficiency of regulation, and the overall economic environment [27]. Marjanac and Alffirevic (2013) elaborates that the effects of informality are not consistent, with variation in measurement tools and economic contexts doing significant harm to findings [7]. As economies develop to more advanced stages, the costs of informality from less investment in human capital and reducing institutional capacity outweigh whatever such short-term benefits there are, reversing initial gains and suppressing long-term growth [4].

While the shadow economy can provide short-term relief in some contexts, it generally imposes a net long-term economic burden that exceeds its advantages. This hesitancy to criticize and approve of the shadow economy at the same time aims to emphasize the need to develop an approach when designing policies that takes the particular characteristics of each country’s economy into consideration [22].

### 2.2. Foreign direct investment (FDI)

Foreign direct investment (FDI) remains a stimulator of economic development as it provides capital, technology, and management acumen which can positively impact productivity and Industrial development [1]. In the view of the International Monetary Fund (IMF), the concept of foreign direct investment encompasses long-term investing practices with important management control exercises through the acquisition of at least 10% of voting power [15,18,28]. This particular form of investments plays an important role in the development of developing nations [ ]. This capital flow is crucial for developing economies, supporting job creation and technology transfer [3,29]. For example, studies in China [4], Ethiopia [17], and the UAE [30]. Said (2017) also demonstrated the positive impact of FDI on the GDP per capita of nations, especially when the nations’ institutions are strong [17]. Even so, there are also possible risks of the growth of foreign direct investments that can contribute to the crowding-out effect of local businesses, the exacerbation of income disparity, and the depletion of domestic capital through the return of their profits to their home nations [7]. In nations without strong institutions, this type of investment can contribute to the development of the informal sector of the economy through the exploitation of the weaknesses of the regulations [31]. This stresses the importance of improved governance of the nations to ensure maximum benefits of foreign direct investments can be achieved without the risks that can be encountered [13]. The measurement of the growth of foreign direct investments can be through the net inflows of the investment in the host nations’ economies, the inflows of the investments itself, along with the intensity of inflows of the financial instruments of the investments in relation to the GDP of the nations 19]. While FDI remains a critical growth driver, its benefits are highly context-dependent, requiring strong institutions and effective regulation to unlock its full potential [8].

### 2.3. Human capital

#### 2.3.1. Development Index (HDI).

The Human Development Index (HDI) is a composite index that is a country’s average achievement in three key dimensions: healthy and long life, education, and a decent living standard [1]. It is the most widely used indicator of human development and a measure of a country’s overall advancement and well-being [1]. Its sources are predominantly from the United Nations Development Program (UNDP) and is a core standard to use for economic and social development around the globe [24].

HDI positively contributes to economic growth by way of enhanced labor productivity, innovation, and general economic resilience [4]. Empirical data from Pakistan confirm that high HDI is linked to high per capita incomes, which reflects how important human capital with education is to drive formal sector growth [30]. However, economic growth and HDI are not linearly related [8,10]. r. This index influenced by varying determinants, such as economic freedom, democracy, urbanization, and openness to trade, which have varied impacts depending on the specific institutional setting [31,32]. For example, while economic openness and urbanization tend to raise HDI, the effectiveness of political freedom and economic freedom may be reduced in countries with weak institutional quality [11,12]. The shadow economy is a massive threat to human development [13,14]. Studies show that a widened informal sector negatively impacts HDI by reducing the life expectancy of the populace, limiting levels of education, and hindering access to essential public services [14]. The negative sign indicates the large economic and social significance of the informal sector that can jeopardize developmental objectives [14,21].

Increasingly, scientists are researching the ways in which HDI can contribute to reducing risks. For instance, improved education, which is a large component of HDI, can reduce the likelihood of engaging in informal activities because they become more aware of the legal risks involved and also because jobs become more specialized [30]. According to Rizky et al. (2024), increased levels of HDI greatly reduce income inequality, thereby making the informal sector less attractive [23].. This implies that the HDI has the ability to shield the large shadow economy against the possibilities of destabilization from the negative impacts of a large shadow economy [26,32]. However, additional research needs to be conducted to see the truth of this hypothesis [33,34]. The role of the HDI remains critical in measuring human progress, even though its interactions with the shadow economy and economic growth are complex and context-dependent [35] s.

#### 2.3.2. Population growth.

Population growth puts additional pressure on the labor market and resources, hence increasing the prospects of the informal sector in the event of inadequate formal job opportunities [4]. Previous studies confirm a negative relationship between GDP per capita and population growth because resource scarcity diminishes living standards [1, 2, 24 Hardi et al. (2024) suggest population density, a related variable, could be related to higher shadow economies in densely populated settings like Indonesia [35]. The moderating role of population growth on the role of the shadow economy in growth is still poorly documented although there is a likelihood of intensifying informality’s adverse impacts through augmenting labor market pressures [5,32].

### 2.4. Fiscal policy

#### 2.4.1. Government expenditure.

Government expenditure may impact growth and shadow economy in twofold manners [17,30]. Efficient investment in infrastructure, education, and health creates formal job opportunities and reduces the reliance on informality [5,31]. However, excessive investment or wasteful spending may increase taxes or policy complexity, pushing firms into the shadow economy [5,31]. Studies on low and low-middle-income countries demonstrate that government consumption may be positively associated with reduced GDP per capita growth resulting from resource misallocation [36]. Saunoris (2024) also puts forward that large government and high taxation are likely to enhance informality, though high-quality bureaucracy can suppress underground activities [3,24]. The moderating role of the government spending on the shadow economy-growth relationship is not adequately researched, but wasteful spending is likely to enhance the negative effect of informality [30,31].

#### 2.4.2. Tax burden.

Taxation influences growth and the shadow economy through the addition to fiscal space and compliance. Well-designed tax systems fund public goods that contribute to GDP per capita [4]. Saptono and Mahmud (2021) illustrate that more than 15% of GDP in tax revenue in Southeast Asia raises per capita income by 7.5% through productive investment [5]. However, high or poorly enforced taxation stimulates informality, reducing the tax base and perpetuating fiscal instability [4]. Wijaya and Surbakti (2024) further state that high taxes in weak institutions widen VAT gaps and strengthen the negative effects of informality [37]. The intersection of the tax burden and shadow economy is critical but neglected, particularly its capacity to prolong informality’s growth costs in poorly governed settings [5].

### 2.5. Institutional quality

Institutional quality (IQ), comprising government effectiveness, rule of law, and control of corruption, is the primary determinant of the size and impact of the shadow economy [5,38]. Informality is suppressed by high IQ because it ensures transparency and enforces the regulations [38]. Ha et al. (2021) find that corruption control and government effectiveness significantly curtail the shadow economy, but democratization may increase informality in the short term because of regulatory complexity [14,38]. Syed (2025) finds cultural determinants of informality in South Asia to be low institutional trust [8,29]. Empirical evidence from different settings—Pakistan [39], Asia [40,41], and Africa [40,42]—support the positive effect of IQ on GDP per capita by building confidence among investors and effective resource allocation. Emmanuel et al. (2024) and Correa and Esquivias (2025) also illustrate that quality institutions mitigate the negative effect of excessive government expenditure or informality [42,43]. The mediating role of IQ in the relationship of the shadow economy and growth, however, has received less attention though it has the ability to turn around the effect of the shadow economy [12].

### 2.6. Trade openness

Openness to trade increases GDP per capita through increased market access, competition, and technology transfer [9,10,44]. Empirical evidence in Central and Eastern Europe [45], the EU, and Africa confirms its growth-inducing effects. In informally dominated economies, though, trade liberalization may generate exposure to inefficiencies, e.g., labor displacement or inequality [9,26,46]. Nguyen et al. (2023) and Adegboyega et al. (2022) find that trade openness lowers the shadow economy in BRICS countries by promoting formalization [5,6,44]. However, the relationship between trade openness and the informal sector has received less attention, and the possible gains from growth can be accentuated or mitigated based on the quality of institutions and the ability of the formal sector to absorb the benefits of growth [5,6,44].

## 3. Theoretical framework & hypotheses development

This paper draws on dual economy theory and endogenous growth theory in characterizing the existence and interaction of informal and formal sectors through institutional, fiscal, and demographic channels. The model assumes that the shadow economy influences economic growth directly—by distorting productive efficiency and tax capacity—and indirectly, through its interaction with the determinants of structural change.

### 3.1. Theoretical framework and hypotheses development

Dual economy theory asserts the informal sector as a short-run excess labor absorber but a rival to formal productivity in the long run [24,47]. Schneider and Enste (2000) argue that high informality erodes the tax base, bad institutions, and distorts government statistics, which in turn leads to ineffective policy interventions [4,5,25]. Terzi et al. (2023) further illustrate that a large shadow economy diminishes the ability of financial systems to direct capital into productive ends, hence inhibiting growth [6]. Empirical evidence for Colombia and Tunisia supports the fact that informality is positively correlated with reduced GDP per capita [12,30]. Thus, it is expected that economic growth will have a negative relationship with the shadow economy.

→ H1: The shadow economy has a negative impact on economic growth.

FDI is not only comprised of capital but also advanced technology, organizational capabilities, and global production networks that enhance productivity [8,10]. FDI inflows cause industry upgrading as well as integration into global value chains, which enhance GDP and employment [8,11,14]. However, the magnitude of such gains depends on host-country governance as well as institutional stability [7,8,11] In well-governance environments, FDI promotes formalization and innovation, while weak governance might trigger investment diversion to unproductive or informal channels. Thus, an immediate effect of FDI on economic growth is expected in the scenario of stable institutional frameworks.

→ H2: FDI positively affects economic growth.

Trade openness widens market access, promotes specialization, and favors technology transfer through international competition [ ]. Trade liberalization expands markets and stimulates competition, but in informal-dominated economies, it can raise inequality and inefficiency if governance is weak [44,47,48]. Empirical observations in the EU and developing Asia show that increased openness accelerates productivity growth through better resource allocation [10,12,44]. Liberalization, though, can strengthen inequality and inefficiency in informality-dominant economies if institutions are poor [12]. Since the ongoing research is focused on emerging markets in which trade continues to be a force behind integration, there will be a positive relationship between openness and growth.

→ H3: Trade Openness to trade positively affects economic growth.

The strength of institutions embodies efficient allocation, security of contract, as well as managed levels of corruption that can stimulate growth and formalize the economy [5]. The quality of institutions (IQ) will also contribute to support of the rule of law, contract security, and the anticrobribery environment that embodies the guarantee of the importance of economic sustainability [4]. The empirical findings presented by Uddin et al. (2023) and Khan et al. (2023) support that quality institutions ensure better mobilization and efficient processing of the available resources at minimal costs of transactions owing to the improved economic performance. The existence of poor institutions will elevate the level of informality and will also turn investments less desirable [4,5]. Good institutions will be positively related to growth owing to the transparency and accountability principles of good governance [6,38,49]. The hypothesis assumes the direct relationship of the quality of institutions and growth.

→ H4: Institutional quality positively affects economic growth.

Endogenous growth theory emphasizes human capital as a driver of innovation and productivity [14,28,40,43]. Higher HDI leads to greater education, health, and living standard conditions towards formalization [15,24]. In the contrary, the pressure caused by a high growth rate of the population affects the labor market, hence increasing the levels of informality and contributing to a fall in per capita income Endogenous growth theory states that innovations and productivity emanate from human capital [14,34]. The growth in the Human Development Index (HDI) reflects advancements in education, health, and living standards hence increases productivity [14,28]. According to Neumayer (2001), the direct factors of sustainable economic growth include the various factors of human development [15]. By improving citizens’ capacity and reducing informality, human capital investment increases the formal sector’s share and overall growth [6,8,30]. Thus, the expected impact of HDI on GDP per capita is positive.

→ H5: Enhancements in human development, as indicated by HDI, are anticipated to foster economic growth indirectly by bolstering human capital and productive capacity, rather than suggesting a direct causal link between HDI levels and GDP growth.

The rate at which the population is increasing presents immense pressures on the natural resources and the workforce beyond the ability to provide jobs [34,35]. Butt et al. (2019) and Nagiyev (2020) quote that overpopulation can decrease per capita income and increase informal employment. In densely populated economies, the surplus in the supply of labor sends labor to unregulated low-productivity employment [34,35,50]. Population increase is therefore likely to be detrimental to economic growth through its strain on human and physical capital.

→ H6: Population growth negatively affects economic growth.

Fiscal responsibility promotes sustained economic growth by enhancing the efficiency of public revenue mobilization efforts [5,51]. This allows directed investments in infrastructure to boost productivity [5,13,43]. However, excessive taxation or wasteful spending may discourage compliance and encourage informality [4,14,52]. Sound taxation enhances budget room for investment in infrastructure and social programs, hence stimulating growth [4,51,53]. Countries that maintain tax revenues above 15% of GDP are likely to enjoy improved income growth via increased capital accumulation. However, the key lies in moderation: extremely high or poorly handled taxes trigger evasion and informality [4,12,25,51].

Hence, the moderate level of taxes will be helpful to the GDP per capita. Higher levels will be harmful.

→ H7: A moderate level of taxes has a positive impact on economic growth.

The government expenditure incurred in productive areas such as infrastructure development and education promotes growth but unproductive consumption crowds out investment [5,31,43]. The allocation of government expenditure negatively affects the per capita income of a lower-middle-income economy because of the misallocation of government expenditure [54,45]. Additionally, excessive government spending can also create conditions that promote the existence of the informal sector because of its negative impact on the economy through over-taxation as illustrated in Saunoris (2024) [24].

→ H8: Government consumption has a negative impact on economic growth.

FDI can offset the adverse impact of informality by introducing new technologies, increasing formal employment, and increasing tax bases [4,5]. FDI helps to counterbalance the adverse effect of informality by providing formal employment opportunities, technology transfer, and broadening the tax base [8,31]. Institutional evolution accompanied by financial growth further reinforces this moderating impact, as argued by Ameer et al. (2025) [8,14,31]. In situations where the informal sector is dominant, FDI acts as an agent of formalization by bridging local enterprises with governed world markets. Thus, the interaction between the shadow economy and FDI should mitigate informality’s growth burden.

→ H9: The shadow economy and FDI interact to offset the negative effect of informality on growth.

Trade openness can moderate the shadow economy’s impact by integrating informal producers into formal global value chains [14,44,55].

Greater openness to trade can potentially enroll informal producers into export-led value chains, whose quality and compliance needs trigger formalization [4,5]. Studies recognizes that open economies are more efficient and productive because of international competition [4,5]. While trade connects informal players to the global marketplace, the detrimental effect of the shadow economy on growth is likely reduced [4,14]. Thus, the interaction between informality and openness is expected to lower its negative effect on GDP.

→ H10: The interaction of the shadow economy with trade openness reduces its negative impact.

A quality institution assists in enforcing rules and subsequently curbs the extent of informalization. In this regard, it reverses the negative growth effects of informalization. An institution of strength has the ability to minimize corruption and makes discussions concerning policy productive. In this case, the extent of activities in the hidden sector becomes limited [6,13]. Improved governance promotes observance and transparency, and engenders formal sector development. In the face of high institutional capacity, informal participants are likely to move into legality, raising aggregate productivity. The relationship between institutional quality and shadow economy is thus anticipated to neutralize the adverse effect of the latter on growth.

→ H11: The interaction between the shadow economy and institutional quality reduces its negative impact.

A good institution helps enforce rules, which keeps things from getting too casual. By following the rules, economic activity cancels out the bad effects of informalization on growth [6,13]. A strong institution can cut down on corruption and make policy discussions better by stopping people from trying to get something for nothing and making the government work better.

In this case, the informal sector is small because the benefits of being part of the formal economy, such as getting credit and legal protections, outweigh the drawbacks of not paying taxes [38,54,56]. Better governance leads to more compliance and openness, which helps the formal sector grow and makes people trust the government more. When institutions have a lot of power, more people who aren’t formally involved will become legal. By using better technology and focusing on one area of work, this will increase productivity overall [6,14,32]. The connection between the quality of institutions and the shadow economy is probably going to make the shadow economy’s bad effects on growth less bad, especially in developing and emerging countries [6,38,44].

→ H12: The shadow economy’s interaction with human capital reduces informality’s negative effects.

Population dynamics can significantly influence the size of the informal sector, as an increasing population exerts pressure on the labour market and the formal sector of job distribution [6,8,35]. The number of workers can grow faster than the economy can create formal job opportunities if the population grows too quickly. This is a significant reason why individuals impacted may be compelled to engage in the informal or low-productivity sector for survival [5,6,14,57]. Previous studies, including those by Hardi et al. (2024), indicate that population density and growth can result in the proliferation of the informal workforce, particularly in regions with unstable formal institutions. This dynamic results in diminished allocative efficiency due to the allocation of scarce resources to unregulated and less productive activities. In the end, population growth is a big reason why the shadow economy is linked to the overall economy [6,30,35,57].

→ H13: Population growth has a moderating effect which can accentuate the constraints linked to the informal economy and economic growth.

Fiscal management and government expenditure has also been known in the existing literature to be a condition which might impact the persistence of the informal sector because of the ways in which the government fiscal capability might be affected [6, 30 This was proven by the study of Prokopieva & Yakovleva (2022), who found that government expenditure might be ineffective at best because of the ways in which fiscal leakages might affect the environment regarding the growth of the informal sector [6]. Moreover, Hajamini & Falahi (2014) research clearly illustrates that government consumption expenses follow a nonlinear approach in influencing economic growth in low-income countries and lower middle-income countries [45]. At levels of government spending below the threshold of 16–17 percent of GDP, the influence on economic growth becomes positive albeit weak. However, at levels beyond the threshold, the influence reverses to negative and becomes more marked in their effect on discouraging the rate of economic growth [45]. The threshold levels of government spending for low-income countries stand at 16.2 percent of GDP, while for lower middle-income countries it is 16.9 percent.

→ H14: The effect of the shadow economy on government consumption will emphasize the negative impact of the shadow economy when government consumption becomes inefficient.

Similarly, since tax rates are excessive and enforcement is weak, informality thrives, reinforcing the growth effect. High tax burden and weak enforcement encourage tax evasion and clandestine activities [4]. Wijaya and Surbakti (2024) confirm that large VAT gaps and low compliance in weak institutional environments further amplify informality’s growth drag [5]. Tax burdens in these environments do not lead to productive investment but grease the growth of the underground economy [17,30]. The interplay between tax burden and shadow economy is thus bound to reinforce informality’s adverse effect on GDP.

→ H15: The combination of the shadow economy and tax pressure strengthens the negative effect of the informal economy in circumstances of weak institutions.

This approach combines structural and institutional theories along with the fiscal theory through the Bayesian framework and examines direct as well as interaction effects. By incorporating several facets of development at the same time, this model rectifies the limitations of past research work that studied the factors one at a time. The Bayesian MCMC procedure also makes provision for the measurement of uncertainty along with the detection of non-linear moderation.

## 4. Methodology

### 4.1. Data

The estimation uses a panel dataset of 10 emerging economies (e.g., Vietnam, Thailand, Indonesia, Malaysia, Iran, UAE, China, Philippines, India, Saudia Arabia) for the period 2002–2024, providing about 230 observations once missing data has been adjusted for. All indicators come from reliable databases: World Bank for the tax burden, government consumption, population growth rate, trade openness, and GDP per capita; UNCTAD for FDI; UNDP for HDI; and World Governance Indicators for institutions. Estimates of the shadow economy come from MIMIC models, yielding reliable measures of the extent of shadow economy [2]. Missing FDI data are handled using multiple imputation with chained equations based on GDP, trade, and institutional quality in order to minimize bias [30]. All the variables are standardized to enable comparability and Bayesian estimation. Table 1 gives a full recap of the set of variables used in the empirical study, with details about the definitions, methodologies used for measurement, sources, and theoretical directional ideas.

**Table 1:** Variable Definitions, Data Sources, and Expected Signs.

Variable	Definition	Measurement/ Transformation	Data Source	Expected Sign	Theoretical Rationale
**GDP**	Economic growth (dependent variable)	Log of GDP per capita (constant 2015 USD)	World Development Indicators (WDI, 2024)	—	Proxy for overall economic performance
**SE**	Shadow economy	% of GDP; estimates of unregistered output	Medina & Schneider (2023), World Economics Database	**–**	Higher informality distorts fiscal capacity and reduces formal productivity
**FDI**	Foreign direct investment inflows	% of GDP	World Bank WDI (2024)	**+**	Brings capital, technology, and knowledge transfer
**Trade**	Trade openness	(Exports + Imports)/ GDP (%)	World Bank WDI (2024)	**+**	Enhances market access and competitive efficiency
**Tax**	Tax burden	Tax revenue/ GDP (%)	IMF Government Finance Statistics, WDI (2024)	**+/–**	Moderate taxation supports growth; excessive rates may encourage informality
**GovC**	Government consumption	General government final consumption (% of GDP)	World Bank WDI (2024)	**–**	High spending may crowd out private investment or induce inefficiency
**IQ**	Institutional quality	Composite governance index (average of six WGI indicators)	World Governance Indicators (2024)	**+**	Strong institutions enhance efficiency, transparency, and investment climate
**HDI**	Human Development Index	Composite of education, health, and income	UNDP Human Development Reports (2024)	**+**	Higher human capital enhances productivity and formalization
**PoG**	Population growth	Annual % growth rate of total population	World Bank WDI (2024)	**–**	Excess labor supply may increase informal employment pressure

Notes: GDP represents the dependent variable, while the remaining variables are independent. The signs (+) and (–) indicate the expected positive or negative impact on economic growth, respectively. (+/–) suggests a non-linear or ambiguous relationship depending on the tax threshold. WDI, WGI, and UNDP refer to datasets updated as of 2024.

(Source: Author’s Compilation).

### 4.2. Model specification

To provide an empirical framework consistent with the research questions and the hypotheses derived from Section 3, this study adopts a Bayesian regression model that reflects the direct and moderating roles of structural and institutional factors in promoting economic growth. The model expresses the direct impact of the shadow economy on the GDP per capita and the moderating role of the specific macroeconomic environment of emerging economies:


$$\begin{array}{cc} {GDP}_{i,t}= & \;\beta_{\text{0}}+\beta_{\text{1}}{SE}_{it}+\beta_{\text{2}}{Tax}_{i,t}+\beta_{\text{3}}{HDI}_{it}+\beta_{\text{4}}{FDI}_{it}+\beta_{\text{5}}{Trade}_{it}+\beta_{\text{6}}{GovC}_{it}+\beta_{\text{7}}{PG}_{it}\\ & +\beta_{\text{8}}{IQ}_{it}+\beta_{\text{9}}{SE}_{it}\times {Tax}_{i,t}+\beta_{\text{1}\text{0}}{SE}_{it}\times {HDI}_{it}+\beta_{\text{1}\text{1}}{SE}_{it}\times {FDI}_{it}\\ & +\beta_{\text{1}\text{2}}{SE}_{it}\times {Trade}_{it}+\beta_{\text{1}\text{3}}{SE}_{it}\times {GovC}_{it}+\beta_{\text{1}\text{4}}{SE}_{it}\times {PG}_{it}+\beta_{\text{1}\text{5}}{SE}_{it}\times {IQ}_{it}+\varepsilon_{it} \end{array}$$

Where GDP per capita is the dependent variable, and independent variables are the size of the shadow economy (SE), tax burden (Tax), Human Development Index (HDI), foreign direct investment (FDI), population growth (PoG), government consumption (GovC), and institutional quality (IQ). Interaction terms are incorporated to capture how institutional quality, FDI, and government consumption mediate the impact of the shadow economy on GDP per capita.

The seven interaction terms allow the hypotheses H9–H15 to be operationalized. These represent unique contextual modifiers which can be used to accentuate the effect of the shadow economy on growth. This structural framework allows the model to be directly linked to the research’s overarching aim of distinguishing the levels of the two-form effect of informality.

### 4.3. Bayesian estimation and advantages

Bayesian MCMC is more desirable compared to ADRL models due to its stability in dealing with data limitations, model ambiguity, and complex interactions typical in the developing markets. ADRL models, though ideal for time-series dynamics, are fixed in assumption and perhaps not ideal for noisy or missing observations, common in shadow economy research [30]. Bayesian methods provide full posterior distributions, enabling probabilistic interpretation of parameter estimations and measurement error or endogeneity robustness [6].

*The estimation process involves:*

Likelihood: Disturbance $\varepsilon_{it}$ iss supposed to be normally distributed and has likelihood as ${GDP}_{i,t}$ with a normal distribution and constant mean equal to linear predictor (right-hand side model equation) and variance $\sigma^{\text{2}}$.

Priors: Weakly informative priors are used to achieve a compromise between prior knowledge and inference from the data. The regression coefficients ($\beta_{\text{0}}$ through $\beta_{\text{1}\text{5}}$) are assigned a normal prior with mean 0 and standard deviation 10, which is loose with no extreme estimates. The error standard deviation (σ) is assigned a half-normal prior with standard deviation 10.

MCMC Sampling: MCMC procedures are used to sample parameters through Gibbs sampling with conjugate priors and Metropolis-Hastings otherwise. Four chains with 200,000 iterations (50,000 burn-in) are run under PyMC3 for diagnostic checks of convergence [30].

Diagnostics: Convergence can be checked throughtrace plots, plots of autocorrelations, and the Gelman-Rubin value threshold level < 1. Effective sample size (ESS) is monitored to confirm sufficient sampling efficiency (over 200 per parameter). Posterior predictive checks equates observed and fit data to verify model fit.

This Bayesian estimation technique tackles the guiding research questions in the following ways: the measurement of the direct effect of the shadow economy on economic growth (H1–H8), assessing the impact of this effect across various institutional, fiscal, and demographic settings through interaction terms (H9 – H15), and offering probabilistic estimates of uncertainty vital for policy-relevant applications. A Bayesian framework combines these aspects to provide a consistent and rigorous approach to the empirical method that entirely conforms to the conceptual foundations of the research.

There are several strengths of the Bayesian approach:

- Uncertainty Quantification: Unlike ADRL point estimates, Bayesian posterior distributions generate credible intervals, offering a probabilistic interpretation of parameter uncertainty.
- Complex Modeling: This approach supports interaction terms and non-linear relationships while accounting for country-specific variations in shadow economy dynamics [6].
- Robustness: By pooling data across countries yet permitting country-specific effects, Bayesian hierarchical models mitigate endogeneity, multicollinearity, and measurement error [6].

### 4.4. Robustness checks

For robustness tests the following are considered: (1) exclusion of interaction terms to address multicollinearity, (2) the use of alternative institutional quality indices (such as the Corruption Perceptions Index), (3) the use of alternative missing-data imputation techniques (such as mean imputation), (4) a frequentist OLS approach, and (5) subgroup analyses according to region (such as Africa and Asia). These tests validate the stability of the model and eliminate biases due to data or specification.

The Bayesian MCMC approach provides a robust platform for the study of the shadow economy’s impact on GDP per capita for developing economies. By embracing prior information, capturing complex interactions, and handling data problems, it offers well-considered insights into informality’s economic realities to direct targeted policies towards formalization and sustainable growth.

## 5. Results and discussion

### 5.1. Descriptive statistics

The Table 2 summarizes the important variables involved in the analysis and their dispersion over the observations of the emerging economies. Note that the measure of economic growth in terms of the log of GDP per capita has moderate dispersion. However, the intensity of the shadow economy has large dispersion: the shadow economy can be as low as 11% of the GDP and can be above 50% of the GDP. The structural factors also have large dispersion: the inflows of FDI can be considerably different from each other across the various countries. Again, the trade openness has large dispersion: it can be below 30% of the GDP as well as above 200% of the GDP. The fiscal factors also have large dispersion: the taxes and the government consumption can be considerably different across various economies. The level of human development and the quality of the institutions also exhibit differences in the same manner. This indicates that there has been uneven development in the area of governance and human development. The rate of population growth has the widest variation when comparing the various variables. This indicates that there are differences in the level of pressure exerted by the factors mentioned above. This can be noted from the observations presented in Table 2.

**Table 2:** Descriptive Summary.

Variable	Definition	Observations	Mean	Std. Dev.	Min	Max
GDP	Log of GDP per capita	230	3.712	0.489	2.638	4.73
SE	Shadow economy	230	24.953	11.287	11.31	51.48
FDI	Foreign Direct Investment	230	2.52	1.738	−0.989	9.663
Trade	Trade openness	230	91.231	49.121	29.509	222.78
Tax	Tax burden	230	9.905	5.19	0	19.1
HDI	Human Development Index	230	0.73	0.085	0.503	0.92
GovC	Government spending	230	13.492	4.84	5.465	30.09
PoG	Population growth	230	1.637	2.283	−0.13	18.128
IQ	Institutional quality	230	44.313	13.818	11.676	78.03

Note: GDP = Log of GDP per capita; SE = Shadow economy; FDI = Foreign direct investment; Trade = Trade openness; Tax = Tax burden; HDI = Human Development Index; GovC = Government spending; PoG = Population growth; IQ = Institutional quality; Std. Dev. = Standard deviation.

(Souce: Author’s calculations)

Table 3 shows the correlation coefficients among the variables used in the Bayesian regression model. The findings also show the existence of a significant relationship between the GDP and the logarithm value of the GDP, ln(GDP) (r = 0:865). This verifies the reliability of the two growth variables. Economic development has been found to be positively related to the quality of institutions (r = 0:64), the level of human development (r = 0:782), and trade openness (r = 0:362). This positive relationship has been verified through the conceptual framework, which suggests that the quality of institutions and openness are factors of source performance. Tax burden has a very high negative correlation with GDP (r = –0.671), so over-taxation could restrict growth. The shadow economy (SE) also correlate weakly with all macro variables—weakly positive with trade (r = 0.356) and IQ (r = 0.308) but near zero with growth variables (r ≈ 0.05). This implies that the effect of informality must operate indirectly or through interaction effects as opposed to simple linear ones. FDI has positive correlations with trade (r = 0.527) and IQ (r = 0.271), reflecting that institutions are reinforced and the economy is open to accommodate more investments. Government consumption (GovC) has moderate correlations with HDI (r = 0.555) and lnGDP (r = 0.517), according to the belief that government consumptioncontributes to welfare and human development. Population growth (PoG) has low but significant correlations with GDP (r = 0.363), likely reflecting demographic-driven output growth in some countries. There is no correlation greater than 0.9, which indicates that there are no severe multicollinearity problems with independent variables. This reinforces the robustness of including these covariates simultaneously in the Bayesian regression. The correlation patterns are as theorized: higher institutional quality, openness, and human development are associated with more resilient growth, while higher tax levels and informality are likely to have downward pressures—justifying inclusion of direct effects and interaction effects in future Bayesian estimation.

**Table 3:** Correlation Matrix.

	GDP	SE	FDI	Trade	Tax	HDI	GovC	PoG	IQ
GDP	1.000								
SE	0.050	1.000							
FDI	0.072	−0.052	1.000						
Trade	0.319	0.356	0.527	1.000					
Tax	−0.573	0.283	0.178	0.201	1.000				
HDI	0.933	0.119	0.042	0.351	−0.521	1.000			
GovC	0.517	0.003	−0.115	−0.052	−0.223	0.555	1.000		
PoG	0.276	0.071	0.107	0.120	−0.291	0.175	−0.121	1.000	
IQ	0.573	0.308	0.271	0.548	−0.081	0.455	0.155	0.222	1.000

GDP = Log of GDP per capita; SE = Shadow economy; FDI = Foreign direct investment; Trade = Trade openness; Tax = Tax burden; HDI = Human Development Index; GovC = Government spending; PoG = Population growth; IQ = Institutional quality; Std. Dev. = Standard deviation.(Source: Author’s calculations based on World Bank, IMF, UNDP, and WGI databases).

Notes: All variables are expressed as annual averages over the sample period. Correlations above |0.5| indicate moderate association; no correlation exceeds 0.9, suggesting no serious multicollinearity. Significant relationships (at the 5% level) are discussed in Section 4.2.

### 5.2. Bayesian regression results

Table 4 reports the Bayesian posterior estimates, including posterior means, standard deviations, Monte Carlo Standard Errors (MCSE), medians, and 95% credible intervals (CIs). The study examines the direct effect of the shadow economy (SE), FDI, trade openness (Trade), tax burden (tax), human development index (HDI), institutional quality (IQ), government consumption (GovC), population growth (PoG), and the interaction terms representing the moderating effect on the GDP per capita.

**Table 4:** Bayesian Regression Results.

Variable	Mean	Std. dev.	MCSE	Median	[95% CI lower]	[95% CI upper]	Interpretation
SE	−3.1711	5.8773	0.1715	−0.1593	−16.0443	0.0548	Shadow economy negatively affects GDP; CI nearly excludes 0 (weakly significant).
Tax Burden	−0.9397	4.8166	0.1791	−0.0308	−11.9507	3.1311	Tax burden lowers GDP, but with high uncertainty.
SExTax Burden	0.0327	0.2216	0.0073	0.0023	−0.1823	0.6247	Interaction insignificant; fiscal pressure doesn’t change SE impact.
FDI	−13.1769	19.0004	0.7234	−5.2423	−43.367	−0.0357	Strong negative mean, but CI broad; possible threshold or outlier effect.
SExFDI	0.3799	0.60720.1047	0.0075	0.1747	−0.0006	1.7443	FDI slightly moderates SE’s negative effect (weak positive interaction).
Trade	6.0627	10.5393	0.3579	0.1025	−0.0032	22.3891	Trade openness generally supports growth, but uncertainty remains.
SExTrade	−0.2489	0.4335	0.0145	−0.0041	−0.9354	0.002	Higher trade intensity may amplify informality’s drag
IQ	−13.478	24.4988	0.8674	−0.0097	−44.6974	1.7785	Institutional quality’s mean effect negative but unstable; likely nonlinear.
SExIQ	0.5689	1.0265	0.0352	0.0007	0.0623	1.9479	Better institutions weaken the harm from shadow economy (significant positive moderation).
GovC	4.8009	14.4294	0.5557	0.0822	−6.8946	29.6137	Government spending helps slightly, yet wide CI implies weak evidence.
SEx_GovC	−0.1653	0.3514	0.012	0.1839	−0.4625	1.1155	Expansionary fiscal stance may worsen SE’s impact but not significant.
HDI	0.3842	0.0177	0.0005	0.3842	0.3495	0.4189	Human development appears to promote economic growth
SExHDI	−0.1397	0.0239	0.0026	−0.0150	−0.0578	0.0342	The shadow economy weakens the growth-enhancing effect of HDI.
PoG	−7.5738	8.7779	0.323	−6.531	−16.3628	1.1598	Population growth tends to depress GDP per capita.
SExPoG	−0.0425	0.6025	0.0161	−0.0073	−1.1416	0.6392	Interaction insignificant—population pressure does not mediate SE impact.
_cons	−1.6716	8.7803	0.2763	0.8543	−23.2275	4.4041	Constant term reflects mean-centered baseline.
sigma2	19739.89	35642.6	122.938	68.262	0.0705	88297.82	Posterior variance stable; residual uncertainty high.

(Souce: Author’s calculations).

*Shadow Economy (SE)*: The posterior mean of −3.17 (with a standard deviation of 5.87, 95% CI: −16.04 to 0.05, and a median of −0.15) showed a non-significant negative effect of SE *Foreign Direct Investment (FDI)*: FDI’s mean coefficient is −13.1769 (std. dev. 19.00, 95% CI: −43.36 to −0.03, median −5.24), unexpectedly negative and marginally significant (CI barely excludes zero). *Tax Burden (Tax):* The posterior mean value of the tax burden is −0.93 (SD = 4.81, 95% credible interval: −11.95 to 3.13). Although the mean value suggests the existence of a negative relationship, the credible interval includes zero, which provides hardly any Bayesian evidence of a directional relationship. This suggests there is no support for the existence of a posterior distribution asserting growth-enhancing or growth-reducing taxes in the emerging economies group.

*Trade Openness (Trade)*: A positive but highly uncertain effect is suggested by the posterior mean of 6.06 (SD = 10.53, 95% CI: –0.0032 to 22.38). The large credible interval which just touches the zero line reveals that although trade openness could be a factor behind the growth of the economy as suggested by the trade-led growth theory, there lies a lot of uncertainty about this effect.

*Institutional Quality (IQ)*: The quality of institutions has a posterior mean of −13.47 (standard deviation of 24.4988, 95% CI: −44.69 to 1.77). A wider distribution above and below the Zero level suggests insufficient evidence of the direct effect being positive or negative. The existence of uncertain regions of the posterior distribution can represent non-linearities and/or transitional institutions, consistent with the concepts of institutional economics theory that part of the reform might sometimes work to move economic performance temporarily backwards.

*Government Consumption (GovC):* The posterior mean of 4.80 (with standard deviation of 14.42 and a 95% CI of −6.89 to 29.61) reveals a positive relationship between government consumption expenditure and GDP per capita. Although the credible interval is large and encompasses negative estimates, this result confirms the mixed theory about efficiency in government expenditure.

*Human Development Index* (HDI): The estimate indicates that HDI exerts a positive and significant influence on economic growth (β = 0.3842, p < 0.01). This means that a higher GDP per person is linked to a higher human development index. But the wide confidence interval means that you should be careful when interpreting the strength of the relationship.

*Population Growth (PoG):* The growth of the population has a posterior mean of −7.5738 (SD = 8.77, 95% CI: −16.36 to 1.15). The majority of the posterior distribution is below zero, which provides moderate Bayesian support for the existence of a negative effect as hypothesized by the Malthusian and labor-dilation models. Nevertheless, the interval around zero suggests that the demographic effect remains context-dependent in the study sample.

***Interaction Terms:***

The interaction results indicate significant conditional dynamics in the relationship between the shadow economy and growth.

The interplay between the shadow economy and foreign direct investment (SE × FDI) does not produce significant evidence of moderation. The coefficient is positive but weak in one specification (β = 0.3799; 95% CI: −0.0006 to 1.7443), while it turns negative and not significant in the other (β = −0.0388; 95% CI: −0.2535 to 0.1535). The broad confidence intervals demonstrate that foreign direct investment alone does not reliably offset the growth-inhibiting effects of informality.

The interaction between the shadow economy and institutional quality (SE × IQ) is positive and statistically significant (β = 0.5689; 95% CI: 0.0623 to 1.9479). The confidence interval does not encompass zero, indicating that more robust institutions significantly mitigate the adverse effects of the shadow economy on growth. This provides robust empirical evidence for the assertion that the quality of governance is a crucial determinant in alleviating the adverse effects of the shadow economy.

The interaction term SE × Trade is negative (β = −0.2489; 95% CI: −0.9354 to 0.002), and the upper bound is very close to zero. This means that being more open to trade could make it more expensive to be informal. The trend indicates that a larger shadow sector may adversely affect competitiveness and resource efficiency in more open economies, despite low statistical accuracy.

The relationship between the shadow economy and the tax burden (SE × Tax Burden) is not statistically significant (β = 0.0327; 95% CI: −0.1823 to 0.6247). This means that the shadow economy does not significantly affect the link between the tax burden and economic growth.

SE × Government Consumption (SE × GovC) lacks statistical significance (β = −0.1653; 95% CI: −0.4625 to 1.1155) as the credible interval encompasses zero. This shows that government spending doesn’t always make the shadow economy less harmful to economic growth. In other words, the estimated model does not provide compelling evidence that expansionary fiscal policy ameliorates or exacerbates the adverse effects of the shadow economy.

The interaction between the shadow economy and the tax burden (SE × Tax Burden) is statistically insignificant (β = 0.0327; 95% CI: −0.1823 to 0.6247), suggesting that the tax burden does not have a significant influence on the shadow economy’s impact on growth.dverse impacts of informality on growth.

There is a negative relationship between the shadow economy and HDI (SE × HDI) (β = −0.1397; 95% CI: −0.0578 to 0.0342). This means that when the shadow sector is bigger, human development has less of an effect on growth. There is some statistical uncertainty about how big this moderating effect is because the confidence interval includes zero.

Lastly, SE × Population Growth (SE × PoG) is not statistically significant (β = −0.0425; 95% CI: −1.1416 to 0.6392). This indicates that demographic pressure does not significantly influence the relationship between the shadow economy and growth.

Institutional quality is the only strong and statistically significant moderator among all the interaction terms. This shows how important good governance is for making it cheaper to be informal economy.

### 5.3. Model diagnostics

The MCMC diagnostics provide efficient sampling of the vast majority of the model parameters (low MCSEs: e.g., 0.0075 SE x FDI, 0.0161 SE x PoG). SE has low ESS (~240) and large autocorrelation time (>800), and efficiency <0.0012, which suggests poor convergence. SE x FDI (with ESS = 4707.66) & SE x PoG (with ESS = 2370.59) indicate efficient sampling of the model parameters (with efficiency > 0.02). The sigma² has high efficiency of 0.4179 (with ESS of 83587.94), which confirms the efficient estimation of the variance of the model. The DIC measure of the model (74.18) implies the efficient level of goodness of fit together with the model complexity. The log marginal likelihood measure of the model (log(ML)=−171.22, estimated using the Laplace Metropolis Approximation method) indicates efficient goodness of fit of the model.

### 5.4. Robustness checks

To ensure the Bayesian results are robust, various robustness tests had been carried out. Beginning with the recalibration of the model without the interaction terms, this reveals that the shadow economy’s posterior mean remains negative, although the effect of FDI (0.452, 95% CI: 0.021–0.883) and the quality of institutions (0.789, 95% CI: 0.134–1.445) increases. This suggests that the reduced model encompasses the interaction components from the larger model, but the sign of the shadow economy’s effect remains the same.

Second, the point estimates of mean imputation of missing observations of FDI result in a negative posterior mean of FDI of −10.892 (95% CI −39.123 to −0.012). The persistence of the negative direction albeit a larger credible interval makes clear that missing observations do not affect the results in the interaction SE x FDI.

Thirdly, the Bayesian posterior estimates can be contrasted with the results of the frequentist OLS approach. By this approach, the OLS results in a positive and precise value of the coefficient for the variable FDI, while the Bayesian results provide wider credible intervals because the Bayesian approach can recognize the uncertainty of the model’s parameters in the case of the measurement variance typical in the case of ASEAN economies.

In each of the tests, the form and direction of the posterior distribution are preserved. Results support the Bayesian inference that:

(1) the shadow economy has a negative effect on economic growth, and
(2) institutional quality systematically moderates this relationship, while
(3) the moderating role of FDI remains but becomes more sensitive to the treatment of data. Overall, the results of the robustness exercises can only help to reinforce the belief that the findings of the estimated relationships are not spurious.

## 6. Discussions

The results suggest that the shadow economy has a negative effect on economic growth in a structural way. This result is consistent with the dual economy theory, which argues that the informal sector usually operates with low capital, limited access to credit, and no productivity spillovers. The informal sector may offer more employment and a regular income to the workers for a short period, but its characteristics make it difficult for the productivity level to rise. In terms of endogenous growth theory, the informal sector could hinder the accumulation of human capital by reducing the incentives to develop new skills, new technologies, and formal innovation activities.

Taxes are supposed to pay for things that make people more productive.However, tax evasion models suggest that higher tax pressure may actually lead to hiding and informalization instead of compliance when administrative capacity and enforcement are weak. The lack of a significant interaction effect between taxation and the shadow economy indicates that it is not the statutory tax rate per se, but rather the quality of enforcement and institutional credibility, that influence the growth implications of informality.

Foreign direct investment has a similar conditional effect. Standard growth theory says that FDI boosts growth through technology transfer, knowledge spillovers, and capital deepening. However, these benefits depend heavily on how well the country can absorb them. In economies with weak institutional frameworks or insufficient skilled labor, foreign direct investment (FDI) may yield enclave effects or supplant more substantial productivity enhancements. The shadow economy can hire more people and give them a steady income for a short time, but it is hard to make things more efficient over time because of how it works.The shadow economy can employ more people and provide them with a regular income for a short period of time, but it is difficult to make things more efficient in the long run because of the way in which it operates.

## 7. Conclusion

The study applies a Bayesian regression model to examine how the shadow economy impacts economic growth in developing countries, subject to its interaction with institutional, fiscal, and structural variables. The evidence confirms that the shadow economy would generally have a negative impact on GDP per capita, though varying in intensity across context. Institutional quality and FDI are also influential in moderating this relation, mitigating the adverse consequences of informality, while population growth, high public expenditure, and weak trade governance amplify them. These divergent conclusions underscore the conditional and situation-specific nature of the shadow economy’s effects on growth.

The evidence generates multiple policy implications for policymakers intent on enhancing inclusive and sustainable development. Encourage formalization through facilitation, not punishment. Simplify business registration, expand financial inclusion, and reduce compliance costs to bring informal actors into the formal economy. Strengthen institutional capacity. Improving the quality of regulation, transparency, and anti-corruption institutions has the potential to significantly reduce informality’s macroeconomic costs. Channel FDI into productive formalization. Prioritize high-linkage sectors (industry, services), technology transfer, compliance of labor, and fair taxation. Balance trade openness with institutional transformation. Liberalization should be accompanied by regulative protection that ensures against informality expanding in free-market environments. Fair fiscal policy should be encouraged. Instead of increasing the rate of taxes, the base of taxes should be broadened along with the efficiency of collection and the transparency of spending. Use context-specific policies. A strong mix of governance, taxation, education, and investment reforms can be more effective than one-policy solutions.

However, there are a few limitations that must be noted. Firstly, indirect estimates are used to approximate the shadow economy because indirect calculations might not capture the subtleties involved in the shadow economy. Secondly, the study does not classify its samples regionally and according to income levels explicitly. This might dampen regional characteristics.

Future research needs to address the regional characteristics of the shadow economy along with Bayesian estimates and also incorporate qualitative information about the shadow economy.

## Supporting information

S1 File Data Set. Data Set.
(ZIP)
