# Supplementary material for: Unveiling the shadow economy in emerging markets
Source: PLoS One. 2026 May 13;21(5):e0347882. doi: 10.1371/journal.pone.0347882 (PMC13170881; doi:10.1371/journal.pone.0347882)
Supplement: S1 File — Data Set. (ZIP) [file pone.0347882.s001.zip › Supporting Information/S1 File. Data Set.pdf]

Data Set

Country name	Country	Year	SE	Taxburden	Unem	GDP	lnGDP	Trade	CPI
Vietnam	1	2002	15.40	13.47	2.12	434.81	2.64	116.70	3.83
Vietnam	1	2003	15.44	14.48	2.25	485.45	2.69	124.33	3.23
Vietnam	1	2004	15.15	14.85	2.14	551.90	2.74	133.02	7.75
Vietnam	1	2005	15.03	16.06	2.09	693.19	2.84	130.71	8.28
Vietnam	1	2006	15.06	15.64	2.09	790.59	2.90	138.31	7.42
Vietnam	1	2007	14.99	15.14	2.03	913.31	2.96	154.61	8.34
Vietnam	1	2008	14.97	15.38	1.93	1158.10	3.06	154.32	23.12
Vietnam	1	2009	15.03	14.19	1.74	1225.85	3.09	134.71	6.72
Vietnam	1	2010	14.99	14.93	1.11	1684.01	3.23	113.98	9.21
Vietnam	1	2011	14.67	16.36	1.00	1953.56	3.29	125.26	18.68
Vietnam	1	2012	14.54	15.44	1.03	2190.23	3.34	123.22	9.09
Vietnam	1	2013	14.66	17.01	1.32	2367.50	3.37	130.85	6.59
Vietnam	1	2014	14.60	15.81	1.26	2558.78	3.41	135.41	4.08
Vietnam	1	2015	14.72	16.14	1.85	2595.23	3.41	144.91	0.63
Vietnam	1	2016	14.66	15.36	1.85	2760.72	3.44	145.41	2.67
Vietnam	1	2017	14.56	14.78	1.87	2992.07	3.48	160.98	3.52
Vietnam	1	2018	14.16	14.91	1.16	3267.23	3.51	164.66	3.54
Vietnam	1	2019	14.36	14.66	1.68	3491.09	3.54	164.70	2.80
Vietnam	1	2020	14.43	14.46	2.10	3586.35	3.55	163.25	3.22
Vietnam	1	2021	14.24	14.30	2.24	3756.49	3.57	186.43	1.83
Vietnam	1	2022	14.17	14.38	1.52	4163.51	3.62	185.73	3.16
Vietnam	1	2023	14.10	19.10	1.61	4100.00	3.61	216.30	3.13
Vietnam	1	2024	14.04	19.10	1.50	4520.00	3.66	222.78	3.07
Thailand	2	2002	51.48	13.47	1.82	2091.18	3.32	114.97	0.70
Thailand	2	2003	50.73	14.48	1.54	2350.85	3.37	116.69	1.80
Thailand	2	2004	50.48	14.85	1.51	2647.26	3.42	127.41	2.76
Thailand	2	2005	50.12	16.06	1.35	2876.25	3.46	137.85	4.54
Thailand	2	2006	49.63	15.64	1.22	3343.79	3.52	134.09	4.64
Thailand	2	2007	49.49	15.14	1.18	3934.69	3.59	129.87	2.24
Thailand	2	2008	49.70	15.38	1.18	4327.80	3.64	140.44	5.47
Thailand	2	2009	50.36	14.19	1.49	4154.19	3.62	119.27	-0.85
Thailand	2	2010	49.52	14.93	0.62	4996.37	3.70	127.25	3.25
Thailand	2	2011	49.33	16.36	0.66	5396.64	3.73	139.68	3.81
Thailand	2	2012	48.79	15.44	0.58	5748.63	3.76	137.67	3.01
Thailand	2	2013	48.56	17.01	0.25	6041.13	3.78	132.46	2.18
Thailand	2	2014	48.68	15.81	0.58	5822.38	3.77	130.91	1.90
Thailand	2	2015	48.45	16.14	0.60	5708.79	3.76	124.84	-0.90
Thailand	2	2016	48.19	15.36	0.69	5854.46	3.77	120.58	0.19
Thailand	2	2017	47.72	14.78	0.83	6436.79	3.81	120.89	0.67
Thailand	2	2018	47.27	14.91	0.77	7124.56	3.85	120.84	1.06
Thailand	2	2019	47.13	14.66	0.72	7628.58	3.88	109.69	0.71
Thailand	2	2020	48.38	14.46	1.10	7001.79	3.85	97.80	-0.85
Thailand	2	2021	47.07	17.00	0.99	7060.90	3.85	117.24	1.23
Thailand	2	2022	46.84	17.00	0.86	7300.00	3.86	133.88	6.08
Thailand	2	2023	46.62	17.00	0.98	7665.00	3.88	123.00	5.39
Thailand	2	2024	46.39	17.00	0.90	8048.25	3.91	127.00	5.85
Philipines	3	2002	42.97	11.67	3.63	1037.17	3.02	83.84	2.72

Philipines	3	2003	42.64	11.67	3.53	1049.39	3.02	87.57	2.29
Philipines	3	2004	42.45	11.36	3.55	1122.86	3.05	87.13	4.83
Philipines	3	2005	41.14	11.92	3.80	1245.29	3.10	83.85	6.52
Philipines	3	2006	41.15	13.13	4.05	1452.22	3.16	80.85	5.49
Philipines	3	2007	40.64	12.96	3.43	1741.60	3.24	73.64	2.90
Philipines	3	2008	40.39	13.03	3.72	1990.36	3.30	67.68	8.26
Philipines	3	2009	40.79	11.70	3.86	1893.28	3.28	60.89	4.22
Philipines	3	2010	40.37	11.64	3.61	2201.78	3.34	66.10	3.79
Philipines	3	2011	40.10	11.85	3.59	2431.20	3.39	60.80	4.72
Philipines	3	2012	40.12	12.31	3.50	2671.78	3.43	57.84	3.03
Philipines	3	2013	39.82	12.74	3.50	2847.57	3.45	55.82	2.58
Philipines	3	2014	39.38	13.02	3.60	2935.93	3.47	57.47	3.60
Philipines	3	2015	39.06	13.02	3.07	2974.30	3.47	59.14	0.67
Philipines	3	2016	38.76	13.09	2.70	3038.15	3.48	61.78	1.25
Philipines	3	2017	38.37	13.59	2.55	3077.43	3.49	68.17	2.85
Philipines	3	2018	38.05	14.05	2.34	3194.67	3.50	72.16	5.31
Philipines	3	2019	37.87	14.49	2.24	3413.85	3.53	68.84	2.39
Philipines	3	2020	39.80	13.95	2.52	3224.42	3.51	58.17	2.39
Philipines	3	2021	38.01	14.10	2.63	3460.54	3.54	63.48	3.93
Philipines	3	2022	37.80	14.60	2.24	3498.51	3.54	72.42	5.82
Philipines	3	2023	37.59	14.10	5.20	4200.00	3.62	66.95	5.97
Philipines	3	2024	37.38	12.00	5.10	4410.00	3.64	68.95	5.85
Malaysia	4	2002	32.24	17.45	3.48	4177.10	3.62	199.36	1.81
Malaysia	4	2003	32.10	15.50	3.61	4454.53	3.65	194.20	1.09
Malaysia	4	2004	31.77	15.20	3.54	4924.34	3.69	210.37	1.42
Malaysia	4	2005	31.28	14.83	3.53	5536.84	3.74	203.85	2.98
Malaysia	4	2006	30.83	14.52	3.32	6137.15	3.79	202.58	3.61
Malaysia	4	2007	30.72	14.30	3.23	7144.00	3.85	192.47	2.03
Malaysia	4	2008	30.68	14.66	3.32	8343.30	3.92	176.67	5.44
Malaysia	4	2009	31.63	14.94	3.66	7167.88	3.86	162.56	0.58
Malaysia	4	2010	30.98	13.33	3.39	8880.15	3.95	157.94	1.62
Malaysia	4	2011	30.88	14.79	3.05	10209.37	4.01	154.94	3.17
Malaysia	4	2012	30.84	15.61	3.10	10601.51	4.03	147.84	1.66
Malaysia	4	2013	30.71	15.31	3.16	10727.67	4.03	142.72	2.11
Malaysia	4	2014	29.56	14.84	2.88	11045.58	4.04	138.31	3.14
Malaysia	4	2015	30.07	14.06	3.10	9699.58	3.99	131.37	2.10
Malaysia	4	2016	29.97	13.55	3.44	9555.65	3.98	126.90	2.09
Malaysia	4	2017	29.58	12.95	3.41	9979.70	4.00	133.16	3.87
Malaysia	4	2018	29.12	12.02	3.30	11073.98	4.04	130.40	0.88
Malaysia	4	2019	28.88	11.94	3.26	11132.10	4.05	123.03	0.66
Malaysia	4	2020	30.49	10.88	4.54	10160.83	4.01	116.79	-1.14
Malaysia	4	2021	29.05	10.70	4.05	11109.27	4.05	134.02	2.48
Malaysia	4	2022	28.87	11.64	3.73	11971.93	4.08	146.66	3.38
Malaysia	4	2023	28.69	12.00	3.50	14420.00	4.16	164.80	3.13
Malaysia	4	2024	28.51	12.00	3.40	15104.00	4.18	169.74	4.03
Indonesia	5	2002	19.72	11.83	6.60	888.90	2.95	59.08	11.90
Indonesia	5	2003	19.84	12.39	6.66	1052.41	3.02	53.62	6.76
Indonesia	5	2004	19.83	12.10	7.30	1136.76	3.06	59.76	6.06
Indonesia	5	2005	19.84	12.40	7.94	1249.40	3.10	63.99	10.45

Indonesia	5	2006	19.72	11.90	7.55	1572.80	3.20	56.66	13.11
Indonesia	5	2007	19.44	12.20	8.06	1840.33	3.26	54.83	6.41
Indonesia	5	2008	19.27	13.31	7.21	2144.39	3.33	58.56	10.23
Indonesia	5	2009	19.34	11.06	6.11	2239.10	3.35	45.51	4.39
Indonesia	5	2010	19.05	10.54	5.61	3094.44	3.49	46.70	5.13
Indonesia	5	2011	18.89	11.16	5.15	3613.80	3.56	50.18	5.36
Indonesia	5	2012	18.75	11.38	4.47	3668.21	3.56	49.58	4.28
Indonesia	5	2013	18.68	11.29	4.34	3602.89	3.56	48.64	6.41
Indonesia	5	2014	18.51	10.84	4.05	3476.62	3.54	48.08	6.39
Indonesia	5	2015	18.49	10.75	4.51	3322.58	3.52	41.94	6.36
Indonesia	5	2016	18.27	10.34	4.30	3558.82	3.55	37.42	3.53
Indonesia	5	2017	18.03	9.88	3.78	3839.79	3.58	39.36	3.81
Indonesia	5	2018	17.89	10.23	4.39	3902.66	3.59	43.07	3.20
Indonesia	5	2019	17.64	9.75	3.59	4151.23	3.62	37.63	3.03
Indonesia	5	2020	17.85	8.31	4.25	3895.62	3.59	32.97	1.92
Indonesia	5	2021	17.52	7.97	3.83	4334.22	3.64	40.20	1.56
Indonesia	5	2022	17.38	7.70	3.55	4788.00	3.68	45.39	4.21
Indonesia	5	2023	17.23	12.00	3.31	5260.00	3.72	51.50	5.19
Indonesia	5	2024	17.09	11.80	5.00	5512.50	3.74	53.04	5.09
India	6	2002	22.81	8.68	8.10	468.84	2.67	29.51	4.30
India	6	2003	22.37	9.11	8.36	543.84	2.74	30.59	3.81
India	6	2004	22.14	9.57	8.53	624.11	2.80	37.50	3.77
India	6	2005	22.27	10.08	8.70	710.51	2.85	42.00	4.25
India	6	2006	21.92	11.13	8.63	802.01	2.90	45.72	5.80
India	6	2007	21.44	12.11	8.54	1022.73	3.01	45.69	6.37
India	6	2008	21.89	10.98	8.35	993.50	3.00	53.37	8.35
India	6	2009	21.97	9.81	8.38	1096.64	3.04	46.27	10.88
India	6	2010	21.46	10.39	8.32	1350.63	3.13	49.26	11.99
India	6	2011	21.41	10.18	8.17	1449.60	3.16	55.62	8.91
India	6	2012	21.30	10.84	8.10	1434.02	3.16	55.79	9.48
India	6	2013	21.04	11.00	8.04	1438.06	3.16	53.84	10.02
India	6	2014	20.95	9.98	7.98	1559.86	3.19	48.92	6.67
India	6	2015	20.64	10.57	7.92	1590.17	3.20	41.92	4.91
India	6	2016	20.38	11.15	7.84	1714.28	3.23	40.08	4.95
India	6	2017	20.30	11.39	7.73	1957.97	3.29	40.74	3.33
India	6	2018	20.12	12.02	7.65	1974.38	3.30	43.62	3.94
India	6	2019	19.99	5.23	6.51	2050.16	3.31	39.91	3.73
India	6	2020	21.70	4.78	10.20	1913.22	3.28	37.80	6.62
India	6	2021	20.20	5.97	7.71	2238.13	3.35	45.67	5.13
India	6	2022	20.09	11.70	7.33	2388.62	3.38	49.23	6.70
India	6	2023	19.97	10.50	4.17	2625.00	3.42	41.20	6.95
India	6	2024	19.86	11.70	3.80	2756.00	3.44	42.43	6.81
China	7	2002	12.89	17.10	4.24	1148.51	3.06	42.75	-0.73
China	7	2003	12.73	17.90	4.58	1288.64	3.11	51.80	1.13
China	7	2004	12.61	18.80	4.49	1508.67	3.18	59.51	3.82
China	7	2005	12.49	8.57	4.52	1753.41	3.24	62.21	1.78
China	7	2006	12.36	9.06	4.43	2099.22	3.32	64.48	1.65
China	7	2007	12.13	9.77	4.35	2693.96	3.43	62.19	4.82

China	7	2008	12.31	10.10	4.59	3468.33	3.54	57.61	5.93
China	7	2009	12.26	10.31	4.72	3832.23	3.58	45.18	-0.73
China	7	2010	12.06	10.21	4.53	4550.47	3.66	50.72	3.18
China	7	2011	12.26	10.31	4.55	5614.39	3.75	50.74	5.55
China	7	2012	12.34	10.26	4.58	6300.58	3.80	48.27	2.62
China	7	2013	12.33	9.91	4.60	7020.39	3.85	46.74	2.62
China	7	2014	12.26	9.68	4.63	7636.07	3.88	44.91	1.92
China	7	2015	11.93	9.38	4.65	8016.45	3.90	39.46	1.44
China	7	2016	11.83	9.12	4.56	8094.39	3.91	36.89	2.00
China	7	2017	11.70	9.42	4.47	8817.05	3.95	37.63	1.59
China	7	2018	11.58	9.05	4.31	9905.41	4.00	37.57	2.07
China	7	2019	11.65	8.49	4.56	10143.86	4.01	35.89	2.90
China	7	2020	11.64	8.09	5.00	10408.72	4.02	34.75	2.42
China	7	2021	11.53	7.97	4.55	12617.50	4.10	37.30	0.98
China	7	2022	11.45	7.70	4.89	12720.22	4.10	38.14	1.97
China	7	2023	11.38	15.00	3.90	13125.00	4.12	36.05	1.96
China	7	2024	11.31	15.00	3.80	13781.00	4.14	37.13	1.92
Iran	8	2002	18.71	4.99	12.80	1910.48	3.28	48.17	14.34
Iran	8	2003	18.27	5.20	11.50	2259.52	3.35	50.68	16.47
Iran	8	2004	17.70	5.20	10.30	2751.79	3.44	51.31	14.76
Iran	8	2005	18.05	6.66	11.81	3226.61	3.51	54.44	13.43
Iran	8	2006	17.82	6.22	11.52	3736.18	3.57	53.17	10.02
Iran	8	2007	16.99	5.94	10.77	4838.00	3.68	49.89	17.34
Iran	8	2008	17.09	6.20	10.63	5623.91	3.75	48.23	25.41
Iran	8	2009	17.45	7.36	12.11	5602.56	3.75	43.70	13.55
Iran	8	2010	17.39	0.00	13.68	6458.57	3.81	43.77	10.09
Iran	8	2011	17.13	0.00	12.49	8201.58	3.91	41.22	26.29
Iran	8	2012	17.55	0.60	12.27	8329.00	3.92	44.09	27.26
Iran	8	2013	17.61	1.00	10.60	6280.68	3.80	47.09	36.60
Iran	8	2014	17.49	1.40	10.68	5757.54	3.76	45.35	16.61
Iran	8	2015	17.94	1.80	11.17	4990.94	3.70	39.42	12.48
Iran	8	2016	17.66	1.80	12.62	5497.24	3.74	40.39	7.25
Iran	8	2017	17.50	2.20	12.23	5758.59	3.76	44.74	8.04
Iran	8	2018	17.74	2.60	12.19	3829.83	3.58	58.57	18.01
Iran	8	2019	18.13	3.00	10.74	3276.75	3.52	50.75	39.91
Iran	8	2020	18.12	3.4	9.69	2746.42	3.44	43.81	30.59
Iran	8	2021	18.07	3.80	10.82	4084.20	3.61	44.37	43.39
Iran	8	2022	18.13	4.20	10.96	4387.83	3.64	51.60	45.90
Iran	8	2023	18.20	7.00	3.90	6300.00	3.80	46.35	49.00
Iran	8	2024	18.26	7.00	3.80	6615.00	3.82	47.74	48.02
Saudi Arabia	9	2002	19.45	5.79	5.27	8380.96	3.92	64.59	0.25
Saudi Arabia	9	2003	18.82	5.78	5.56	9321.80	3.97	69.83	0.61
Saudi Arabia	9	2004	18.27	5.77	5.82	10935.02	4.04	75.08	0.52
Saudi Arabia	9	2005	17.92	5.76	6.05	13462.76	4.13	81.95	0.48
Saudi Arabia	9	2006	18.03	5.76	6.25	14848.60	4.17	89.94	2.21
Saudi Arabia	9	2007	17.88	5.75	5.73	15756.19	4.20	94.86	4.17
Saudi Arabia	9	2008	17.19	5.75	5.08	18944.86	4.28	96.10	9.87
Saudi Arabia	9	2009	18.06	5.73	5.38	15064.63	4.18	84.86	5.06
Saudi Arabia	9	2010	17.71	2.53	5.55	17958.95	4.25	82.55	5.34

Saudi Arabia	9	2011	17.13	2.31	5.77	22441.57	4.35	84.86	5.83
Saudi Arabia	9	2012	16.81	2.52	5.52	24069.20	4.38	82.85	2.87
Saudi Arabia	9	2013	17.03	2.71	5.60	23945.51	4.38	81.92	3.53
Saudi Arabia	9	2014	17.13	2.73	5.72	23862.80	4.38	79.56	2.24
Saudi Arabia	9	2015	17.18	3.26	5.60	20442.37	4.31	69.50	1.21
Saudi Arabia	9	2016	16.99	3.28	5.60	19930.41	4.30	59.91	2.07
Saudi Arabia	9	2017	17.26	3.26	5.89	20910.48	4.32	61.81	-0.84
Saudi Arabia	9	2018	17.23	8.29	6.03	24175.58	4.38	61.96	2.46
Saudi Arabia	9	2019	17.36	7.00	5.64	23405.71	4.37	60.20	-2.09
Saudi Arabia	9	2020	18.82	8.24	7.45	20398.06	4.31	49.71	3.45
Saudi Arabia	9	2021	17.58	9.67	6.69	24160.68	4.38	57.14	3.06
Saudi Arabia	9	2022	17.60	7.77	5.64	25000.00	4.40	63.51	2.47
Saudi Arabia	9	2023	17.63	5.60	4.00	25200.00	4.40	77.25	2.74
Saudi Arabia	9	2024	17.66	5.60	3.90	26460.00	4.42	79.56	2.68
UAE	10	2002	27.15	0.38	2.65	30221.97	4.48	93.07	2.80
UAE	10	2003	26.90	0.33	2.75	32607.37	4.51	102.30	3.10
UAE	10	2004	26.82	0.43	2.90	37017.74	4.57	116.62	4.90
UAE	10	2005	27.05	0.71	3.12	42190.55	4.63	119.55	6.20
UAE	10	2006	27.14	0.85	2.93	45339.59	4.66	119.47	9.30
UAE	10	2007	27.67	0.97	2.86	43918.38	4.64	136.80	11.10
UAE	10	2008	28.38	0.99	2.72	45140.77	4.65	148.51	12.25
UAE	10	2009	29.48	1.08	2.67	31722.59	4.50	153.46	1.56
UAE	10	2010	29.68	1.12	2.46	34165.91	4.53	138.89	0.88
UAE	10	2011	29.53	0.31	2.28	40893.02	4.61	147.39	0.88
UAE	10	2012	29.37	0.35	2.17	44386.79	4.65	159.97	0.66
UAE	10	2013	29.38	0.36	2.03	45729.61	4.66	161.10	1.10
UAE	10	2014	29.11	0.35	1.90	46865.96	4.67	164.03	2.35
UAE	10	2015	29.16	0.06	1.76	41525.14	4.62	169.48	4.07
UAE	10	2016	29.10	0.04	1.64	41054.54	4.61	170.90	1.62
UAE	10	2017	29.42	0.07	2.46	43063.97	4.63	172.80	1.97
UAE	10	2018	29.13	0.06	2.24	46722.27	4.67	157.92	3.07
UAE	10	2019	29.39	0.97	2.33	45376.17	4.66	167.38	-1.93
UAE	10	2020	29.82	0.68	4.29	37629.17	4.58	166.57	-2.08
UAE	10	2021	29.33	0.54	3.11	44315.55	4.65	163.40	-0.01
UAE	10	2022	29.32	0.57	2.75	53757.86	4.73	150.00	4.83
UAE	10	2023	29.32	0.62	2.2	46200	4.664642	154.5	3.33
UAE	10	2024	29.31	0.67	2.1	48510	4.685831	159.13	3.26

FDI	HDI	GovC	PoG	IQ
3.99	0.61	6.23	1.03	32.40
3.67	0.62	6.32	1.03	32.44
3.54	0.63	6.39	1.02	31.40
3.39	0.63	5.47	1.00	36.03
3.62	0.64	5.53	0.97	34.10
8.65	0.65	5.55	0.96	34.32
9.66	0.65	5.63	0.98	33.58
7.17	0.66	5.78	1.03	34.85
5.43	0.66	10.38	1.07	33.08
4.30	0.67	9.98	1.07	34.61
4.28	0.67	10.45	1.07	35.00
4.16	0.68	10.92	1.08	36.89
3.94	0.68	10.32	1.07	36.42
4.93	0.68	10.65	1.04	39.46
4.90	0.69	10.40	1.01	41.38
5.01	0.69	10.13	0.97	40.82
5.00	0.70	9.75	0.93	40.98
4.82	0.70	9.58	0.90	41.47
4.56	0.71	9.48	0.91	42.00
4.28	0.70	9.61	0.84	41.15
4.38	0.73	8.98	0.73	41.41
4.70	0.70	10.20	0.89	45.90
4.96	0.71	20.40	0.89	46.80
2.49	0.68	13.17	0.90	59.52
3.44	0.69	12.93	0.86	56.84
3.39	0.70	13.11	0.82	52.47
4.34	0.71	13.65	0.78	50.85
4.02	0.71	13.50	0.75	43.61
3.28	0.72	13.93	0.76	43.74
2.94	0.73	14.34	0.75	42.32
2.28	0.73	15.98	0.72	42.84
4.32	0.74	15.80	0.67	42.48
0.67	0.74	16.14	0.65	43.71
3.24	0.75	16.35	0.64	44.02
3.79	0.75	16.36	0.61	43.70
1.22	0.78	16.92	0.55	42.29
2.22	0.78	17.12	0.48	42.37
0.84	0.79	16.87	0.44	43.17
1.82	0.79	16.30	0.41	43.97
2.71	0.80	16.17	0.32	43.48
1.01	0.80	16.17	0.25	45.73
-0.99	0.80	17.80	0.24	44.16
2.90	0.80	18.26	0.18	43.56
2.06	0.72	17.73	0.13	45.79
1.50	0.80	18.68	0.19	56.10
2.75	0.72	17.54	1.48	48.89
2.10	0.65	10.27	2.06	42.73

0.57	0.65	9.93	2.02	40.07
0.62	0.66	9.14	1.99	35.52
1.55	0.66	8.87	1.94	40.46
2.12	0.66	9.07	1.88	36.53
1.87	0.66	9.23	1.87	37.77
0.74	0.67	8.81	1.87	37.07
1.17	0.67	9.83	1.84	36.38
0.51	0.67	9.72	1.80	35.41
0.86	0.68	9.71	1.78	37.62
1.23	0.69	10.79	1.74	41.02
1.32	0.69	10.82	1.69	43.38
1.93	0.70	10.56	1.62	45.37
1.84	0.70	10.91	1.67	43.92
2.60	0.70	11.26	1.77	40.33
3.12	0.70	11.32	1.76	40.72
2.87	0.71	12.04	1.70	39.76
2.30	0.72	12.47	1.66	39.91
1.89	0.71	15.26	1.63	39.69
3.04	0.70	15.58	1.49	37.77
2.28	0.71	15.01	1.46	40.19
2.62	0.72	17.37	14.90	47.94
2.75	0.72	17.54	1.48	48.89
3.17	0.72	12.96	2.52	63.50
2.92	0.73	12.97	2.44	64.45
3.51	0.74	12.58	2.37	63.05
2.73	0.73	11.47	2.30	64.07
4.73	0.74	11.17	2.23	60.74
4.69	0.75	11.57	2.18	59.87
3.28	0.76	11.50	2.09	56.49
0.06	0.76	13.05	1.98	55.84
4.27	0.77	12.58	1.76	60.40
5.07	0.77	13.27	1.61	59.25
2.83	0.78	13.84	1.62	59.32
3.49	0.79	13.72	1.59	61.37
3.14	0.79	13.33	1.55	65.35
3.27	0.80	13.09	1.50	62.30
4.47	0.80	12.56	1.46	60.71
2.94	0.81	12.19	1.42	59.75
2.31	0.81	11.97	1.32	65.06
2.51	0.81	11.65	1.24	63.79
1.20	0.81	12.96	1.20	63.72
5.42	0.80	12.71	1.12	61.45
3.62		11.66	1.08	63.63
2.00	0.70	20.20	0.89	45.90
2.53	0.81	14.99	0.39	62.42
0.07	0.61	7.26	1.37	24.59
-0.25	0.62	8.13	1.34	23.49
0.74	0.62	8.32	1.27	25.62
2.92	0.63	8.11	1.26	27.09

1.35	0.64	8.63	1.30	31.12
1.60	0.64	8.35	1.31	34.63
1.83	0.65	8.42	1.30	36.17
0.90	0.66	9.59	1.27	34.84
2.03	0.66	9.01	1.25	34.75
2.30	0.67	9.06	1.26	35.34
2.31	0.68	9.25	1.26	38.80
2.55	0.68	9.52	1.21	40.38
2.82	0.69	9.43	1.16	43.67
2.30	0.70	9.75	1.11	41.52
0.49	0.70	9.53	1.06	44.79
2.02	0.70	9.12	1.01	45.60
1.81	0.71	9.02	0.97	45.05
2.23	0.72	8.81	0.94	44.98
1.81	0.71	9.66	0.84	44.91
1.79	0.71	9.25	0.69	45.80
1.62	0.71	7.66	0.64	46.98
2.20	0.72	15.45	1.09	51.00
2.31	0.72	15.60	1.08	52.02
1.01	0.50	11.31	1.78	43.04
0.61	0.52	10.88	1.72	44.79
0.77	0.53	10.40	1.67	43.78
0.89	0.53	10.37	1.60	46.32
2.13	0.54	9.80	1.52	46.67
2.07	0.55	9.86	1.47	45.14
3.62	0.56	10.54	1.42	45.26
2.65	0.57	11.46	1.39	43.34
1.64	0.58	11.01	1.38	42.99
2.00	0.59	11.08	1.36	42.63
1.31	0.60	10.68	1.33	40.97
1.52	0.61	10.30	1.30	41.21
1.70	0.62	10.44	1.24	41.65
2.09	0.63	10.43	1.19	45.18
1.94	0.64	10.31	1.19	45.74
1.51	0.64	10.77	1.16	46.20
1.56	0.65	10.82	1.09	47.47
1.78	0.65	11.00	1.03	46.93
2.41	0.64	11.61	0.96	47.17
1.42	0.63	11.19	0.80	46.43
1.46	0.63	10.35	0.68	47.91
1.99	0.64	13.93	0.79	40.80
2.09	0.65	14.07	0.79	41.61
3.61	0.60	16.07	0.67	32.59
3.49	0.62	15.31	0.62	34.87
3.48	0.63	14.63	0.59	34.38
4.55	0.64	14.80	0.59	34.24
4.51	0.65	14.82	0.56	34.19
4.40	0.66	14.63	0.52	34.84

3.73	0.67	14.49	0.51	36.53
2.57	0.68	14.75	0.50	36.16
4.00	0.69	14.59	0.48	34.07
3.71	0.70	15.24	0.55	35.39
2.83	0.71	15.76	0.68	34.69
3.04	0.72	15.88	0.67	35.32
2.56	0.73	15.82	0.63	37.80
2.19	0.73	16.22	0.58	38.10
1.56	0.74	16.36	0.57	38.68
1.35	0.75	16.32	0.61	41.62
1.69	0.76	16.54	0.47	42.11
1.31	0.76	16.77	0.35	41.37
1.72	0.76	17.13	0.24	42.67
1.93	0.77	15.81	0.09	43.06
1.00	0.77	16.01	-0.01	41.30
0.52	0.77	16.16	0.09	66.30
0.55	0.77	16.32	0.09	67.62
2.74	0.70	11.72	0.97	26.15
1.87	0.70	11.42	0.93	26.44
1.60	0.71	11.24	1.62	26.26
1.28	0.72	12.08	1.61	23.73
0.87	0.73	12.47	1.55	20.79
0.58	0.73	9.48	1.45	18.38
0.48	0.74	10.05	1.37	16.76
0.72	0.74	11.18	1.36	15.10
0.75	0.75	10.80	1.40	14.37
0.68	0.75	13.20	1.28	16.39
0.72	0.77	13.33	1.28	16.78
0.62	0.77	12.97	1.46	16.00
0.46	0.77	12.82	1.90	18.19
0.50	0.78	15.57	2.26	21.23
0.74	0.79	15.78	1.84	22.77
1.03	0.79	16.06	1.43	21.50
0.72	0.79	15.75	1.31	17.79
0.53	0.78	15.30	1.10	15.79
0.56	0.78	14.43	0.84	11.73
0.40	0.77	14.23	0.72	11.68
0.36	0.77	12.73	0.71	11.91
0.21	0.77	17.67	0.69	35.70
0.22	0.78	17.85	0.69	36.41
-0.32	0.75	25.95	2.40	44.17
-0.27	0.76	24.48	2.30	45.15
-0.13	0.77	22.86	2.18	39.62
3.68	0.78	21.34	3.06	41.97
4.85	0.79	22.04	3.96	40.22
5.85	0.79	20.66	3.93	41.26
7.59	0.80	17.70	3.85	43.54
8.50	0.81	22.19	3.74	42.84
5.53	0.82	20.20	3.21	45.41

2.41	0.83	19.23	2.48	39.64
1.64	0.84	19.81	2.20	44.21
1.18	0.85	22.23	2.12	44.14
1.05	0.85	25.71	2.02	45.55
1.22	0.86	29.32	1.92	42.83
1.12	0.86	25.01	2.01	46.18
0.20	0.86	23.53	2.30	44.68
0.50	0.87	23.60	2.38	44.54
0.54	0.87	23.40	2.28	44.06
0.74	0.87	27.96	0.47	44.69
2.21	0.88	23.80	-0.13	46.96
0.71	0.88	20.83	1.27	49.56
2.94	0.87	24.44	0.02	71.40
3.08	0.88	24.68	1.48	72.82
0.09	0.80	10.15	5.06	71.22
3.42	0.81	9.75	4.83	64.43
6.77	0.82	8.75	4.61	66.17
6.03	0.82	7.87	6.96	64.26
5.77	0.83	6.92	13.48	64.64
5.50	0.83	6.73	18.13	65.37
1.60	0.83	7.30	17.40	63.83
0.45	0.83	10.23	13.42	65.20
2.93	0.84	9.68	5.94	63.00
1.98	0.84	9.67	1.10	65.34
2.49	0.85	9.48	1.04	66.45
2.44	0.85	10.71	1.00	68.03
2.67	0.86	10.64	0.96	69.92
2.31	0.87	12.02	0.91	69.56
2.60	0.87	12.11	0.86	69.66
2.65	0.90	13.05	0.82	69.08
2.43	0.91	11.69	0.79	69.65
4.28	0.92	12.26	0.78	69.15
5.69	0.91	14.45	0.82	68.87
4.98	0.91	20.00	0.83	68.82
4.48	0.91	24.20	0.81	70.28
4.3	0.91	29.79	0.89	76.5
4.52	0.92	30.09	0.89	78.03
